# The Effect of CmLOXs on the Production of Volatile Organic Compounds in Four Aroma Types of Melon (*Cucumis melo*)

**DOI:** 10.1371/journal.pone.0143567

**Published:** 2015-11-24

**Authors:** Yufan Tang, Chong Zhang, Songxiao Cao, Xiao Wang, Hongyan Qi

**Affiliations:** Key Laboratory of Protected Horticulture of Ministry of Education and Liaoning Province, College of Horticulture, Shenyang Agricultural University, Shenyang City, Liaoning Province, People’s Republic of China; University of Tsukuba, JAPAN

## Abstract

Lipoxygenases (LOXs) play important role in the synthesis of volatile organic compounds (VOCs), which influence the aroma of fruit. In this study, we elucidate that there is a positive relationship between LOXs activity and VOC production in melon (*Cucumis melo*), and *CmLOX* genes are involved in fruit aroma generation in melon. To this end, we tested four aroma types of melon that feature a thin pericarp: two aromatic cultivars of the oriental melons (*C*. *melo* var. *makuwa* Makino), ‘Yu Meiren’ (YMR) and ‘Cui Bao’ (CB); a non-aromatic oriental pickling melon (*C*. *melo* var. conomon), ‘Shao Gua’ (SHAO); and a non-aromatic snake melon (*C*. *melo L*. *var*. *flexuosus Naud)*, ‘Cai Gua’ (CAI). A principal component analysis (PCA) revealed that the aromas of SHAO and CAI are similar in nature because their ester contents are lower than those of YMR and CB. Ethyl acetate, benzyl acetate, (*E*, *Z*)-2, 6-nonadienal and menthol are four principal volatile compounds that affect the aromatic characteristics of these four types of melons. The LOX activity and total ester content in YMR were the highest among the examined melon varieties. The expression patterns of 18 *CmLOX* genes were found to vary based on the aromatic nature of the melon. Four of them were highly expressed in YMR. Moreover, we treated the fruit disks of YMR with LOX substrates (linoleic acid and linolenic acid) and LOX inhibitors (*n*-propyl gallate and nordihydroguariaretic acid). Substrate application promoted LOX activity and induced accumulation of hexanal, (2*E*)-nonenal and straight-chain esters, such as ethyl acetate. In contrast, LOX inhibitors decreased the levels of these compounds. The effect of *CmLOX*s in the biosynthesis of esters in melons are discussed.

## Introduction

The melon (*Cucumis melo*) is an important alternative model plant for studying fruit ripening characteristic, such as aromatic traits [1]. Among the key sensory attributes of ripening fruit, the aroma plays a particularly distinctive role in determining consumers’ product preferences [2–4]. Most volatile aroma compounds are produced during the maturation period, and emission of volatile compounds is considered a sign of fruit maturity [5,6]. To date, greater than 2000 types of volatile compounds have been detected in various plants, including apples, strawberries, mangoes, tomatoes and peaches [7–11]. The volatile aroma compounds of melons have been thoroughly investigated, and approximately 240 volatile compounds have been identified in different melon varieties, including volatile aldehydes, alcohols, and an especially large number of esters [12–15]. The formation of aroma compounds in melons strongly depends on the melon variety [16]. Climacteric melons exhibit greater aroma intensity and contain significantly higher levels of volatile esters than non-climacteric melons [17,18]. In highly aromatic melon varieties, most volatile compounds are esterified, and their accumulated levels are usually higher than those in less aromatic varieties [19,20]. Hence, esters, which include branched-chain esters and straight-chain esters, are the most abundant aroma-relevant volatile compounds and considered key contributors to the unique aroma of melons[21–25]. Esters are produced from primary metabolites via two different primary pathways [26]. Branched-chain esters are predominantly produced via the amino acid pathway [27]. Whereas, straight-chain esters are synthesized from the lipoxygenase (LOX) pathway [28,29].

LOX (EC 1.13.11.12) is a type of non-heme iron-containing dioxygenase that exhibits regional and stereo specificity [30,31]. The enzymatic reaction of LOX causes polyunsaturated fatty acids (PUFAs), such as linoleic acid (LA) and linolenic acid (LeA), to generate hydroperoxides (HPOs) [30,32]. HPOs are catalyzed by hydroperoxide lyase (HPL) to produce aldehydes, which are catalyzed by alcohol dehydrogenases (ADHs) to produce alcohols. The final step in ester biosynthesis is catalyzed by alcohol acyl-transferase (AAT) [33–35]. LOX is the first key enzyme in ester biosynthesis. LOX appears to play a key role in the production of volatile esters in peaches, apples and frozen melons [36–39]. A decrease in the LOX activity affects the synthesis of the main aroma compounds in the waxberry [40]. LOXs are widely distributed in plants and encoded by multiple gene families and there are many LOX genes involved in aroma production in plants. In the tomato, *Tomlox*C is involved in the generation of fatty acid-derived C6 short-chain flavor compounds [41]. In kiwifruit, *AdLox1* and *AdLox5* seem to be associated with fruity aroma ester release, and specific LOX genes are related to fruit aroma generation [42]. In tobacco, *NaLOX2* is involved in the biosynthesis of volatile compounds [43]. Therefore, we hypothesized that the enzymatic reaction of LOX is the critical step at which the production of aroma volatile compounds is regulated in melons. Furthermore, we hypothesized that specific *CmLOX* genes are related to the generation of fruit aroma in melon.

As previously described in detail, eighteen candidate LOX genes (*CmLOX01-18*) have been identified in the melon genome. The expression patterns of members of the LOX gene family differ. Six genes (*CmLOX01–06*) belong to the type I 13-LOX family, eight genes (*CmLOX08*, *CmLOX10–16* and *CmLOX18*) belong to the type II 13-LOX family and two (*CmLOX07* and *CmLOX09*) have been proposed to exhibit I 9-LOX activity. *CmLOX01*, *CmLOX03*, *CmLOX16* and *CmLOX18* are expressed at a high level in the oriental melon starting 30 days after pollination (DAP) until 40 DAP. These genes are putatively associated with several late events, such as the development of fruity aroma via ester production in fruit ripening [44]. However, the relative contribution of each member of the LOX gene family in volatile ester production, remain uncharacterized. To identify the effect of LOX on the production of volatile aroma compounds in melons and which *CmLOX* genes involve in fruit aroma generation in melon, two experiments were performed: first, the production of volatile organic compounds (VOCs), LOX activity and gene expression were examined in ripe fruits of four aromatic melon types. Second, a fruit disk experiment was conducted to investigate the effects of LOX substrates and inhibitors on LOX activity, VOC production and *CmLOX* gene expression in melons, to see whether LOX and *CmLOX* genes are stimulated by substrates and inhibitors.

## Materials and Methods

### Plant materials

Different aromatic melon varieties were examined in this study, including the highly aromatic oriental melon (*C*. *melo* var. *makuwa* Makino) cultivar ‘Yu Meiren’ (YMR), the less-aromatic oriental melon (*C*. *melo* var. *makuwa* Makino) cultivar ‘Cui Bao’ (CB) and two nonaromatic varieties of oriental melon, ‘Shao Gua’ (SHAO), which is a pickling melon (*C*. *melo* var. conomon), and ‘Cai Gua’ (CAI), which is a snake melon (*C*. *melo L*. *var*. *flexuosus Naud)*. Melon seedlings were individually grown in pots (volume of 25 L and soil:peat:compost ratio of 1:1:1) in a greenhouse at Shenyang Agricultural University in Shenyang, China, from March through June in 2013 and 2014. Eighty plants of each type of melon were grown in 2013, and 100 plants of YMR were grown in 2014. Flowers were hand-pollinated and tagged on the day of bloom, and only two fruits were allowed to develop for each plant. Fruits of each type of melon (YMR, CB, SHAO and CAI) were harvested at commercial maturity and various DAP in 2013, i.e., 32, 32, 28 and 28 DAP, respectively. YMR fruits at 32 DAP were harvested in 2014 and used in the fruit disk experiment.

### Firmness, soluble solids content (SSC) and rind color

The firmness of the entire fruit was non-destructively measured using a method adapted from Tijskens [45]. A fruit hardness tester (FHM-1, Takemura, Japan) calibrated with a 1 kg weight and equipped with a 12 mm diameter probe was used. Seven readings were obtained for each fruit at two pared surfaces on the equator and recorded in units of N/cm^2^ after a controlled deformation. The SSC and the rind color were determined based on a method adapted from Liu [25]. The SSC of each melon was also measured by dropping the extracted juice from the equatorial region of flesh tissue onto a digital refractometer (DBR45, Huixia, Fujian, China). The rind color was detected using a chromatic color difference meter (CR—400/410, Konica Minolta, Japan); corresponding points on the fruit rind equator were detected, and seven values were recorded. Among these values, L* represents the brightness of the rind, which directly correlates with the fruit luster; a* represents the red/green ratio: higher positive values indicate red fruit, whereas negative values indicate green fruit; and b* represents the yellow/blue ratio: higher positive values indicate yellow fruit, whereas negative values indicate blue fruit. Each experiment was performed in triplicate.

### LOX activity assay

The LOX activity was determined as previously described by Chen and Zhang but with some modifications [42,46]. The flesh tissues were sampled from the equatorial region of the fruit. Approximately 3 g of flesh were ground in 8 mL of 50 mM sodium phosphate buffer (pH 7.0) using a mortar and pestle. The mixture was spun down at 15,000 g for 15 min at 4°C, and the supernatant was then collected to serve as the crude enzyme source for analyzing the LOX activity. Specifically, the supernatant was treated with the substrate linoleic acid, and the consequent LOX activity was measured by continuously monitoring the increase in the absorbance at 234 nm on a CARY 100 scan ultraviolet (UV)/visible spectrophotometer (Varian, USA). This absorbance increases due to the formation of conjugated diene structures during the oxidation of polyunsaturated fatty acids. The reaction was started by addition of 0.2 mL of crude fruit extract to 25 μL of substrate containing 2.775 mL of 0.1 M sodium acetate buffer (pH 6.0), which served as the reaction buffer. One unit (U) of LOX activity is defined as the amount of enzyme that catalyzes the formation of 1 μmol of product per minute, and the measured LOX activity is expressed as the specific activity (mU/mg protein). Each sample consisted of three replicates, and each measurement was repeated three times.

### Protein content

The protein concentration of enzyme extracts was determined using Coomassie brilliant blue G-250 with the method described by Bradford for protein assay with modifications (Bio-Rad Protein Assay kit, Bio-Rad, USA) according to manufacturer’s instructions [47], using bovine serum albumin (BSA) as a standard.

### Volatiles analysis

The gas chromatography–mass spectrometry (GC–MS) analysis methodology based on the procedures described by Obando-Ulloa and Liu was used [17,25]. One hundred gram of frozen melon flesh samples were thawed at room temperature for 30 min. Fresh juice was squeezed from flesh with a juicer (JYL-C05, China), and juice samples were taken by filtering juice through a glass funnel and four layers of cheesecloth. Then 3.5 g sodium chloride (analytical grade) and an internal standard (50μL of 1-octanol, 59.5 mg/L, 0.5%, v/v, Aladdin Chemistry, China) were added to 10 mL supernatant of the juice. The mixture was homogenized completely and poured into a 20-mL glass vial (Thermo, USA). The vials were sealed using a crimp-top cap with silicone/aluminium septa seals (20 mm, Thermo, USA), and heated at 40°C in a water bath. Then aroma volatiles were extracted from the headspace for 30 min with a SPME fibre (100 μm polydimethylsiloxane) with 1 cm long standard needle for manual operation (Supelco, 57347-U, Bellefonte, PA, USA), which was previously preconditioned at 250°C for 30 min in the gas chromatography injection port. After extraction, the SPME device was manually inserted into the split/split-less injector port of a GC–MS system (Trace GC-Ultra-ITQ 900, Thermo Scientific, Waltham, MA 02454), and held in split-less mode at 250°C to desorb the aroma volatile compounds within 3 min. The injector was mounted on a Trace GC-Ultra^™^ with an injection liner (105 mm× 3 mm inner diameter (i.d.), Thermo, USA). The volatile components were separated into a 30 m×0.25 mm i.d. ×0.25 μm thickness capillary column (Thermo TR-5MS, USA). During desorption, the injector was first held at 36°C for 3 min and then maintained for a further 2 min. The temperature was increased to 60°C at a rate of 12°C /min, further increased to 140°C at a rate of 6°C /min, and finally increased to 250°C at a rate of 20°C /min. Subsequently, the temperature was maintained at 250°C for 8 min using helium as a carrier gas (1.0 mL/min). The transfer line to the mass spectrometer was maintained at 270°C. The mass spectra were obtained via electron ionization (EI) at 70 eV, and a spectral range of 40–450 m/z was used. The detector was operated at 230°C in full-scan mode with data acquisition and ion mass captured between 33 and 350 amu. The total analysis time was 40 min. Each sample consisted of three replicates, and each measurement was repeated three times. The chromatographic data regarding aroma compounds were analyzed using Xcalibur^®^2.0 (a Thermo Electron mass spectrometry data system). Compounds were identified by comparing their mass spectra with those included in the National Institute for Standards and Technology (NIST05, search version 2.0) databank and based on the retention times. In addition, the Chemical Abstract Service (CAS) numbers of the volatiles reported in the NIST98 Database were used to obtain their corresponding International Union of Pure and Applied Chemistry (IUPAC) nomenclature. Quantitative analysis of individual compounds was performed using the peak area of internal standard 1-octanol (59.5 ppm) as a relative value. Only the results of SI (similarity index) >800 and RSI (reverse similarity index) >800 have been listed.

### RNA isolation and real-time quantitative RT-PCR analysis

The real-time quantitative RT-PCR (qRT-PCR) analysis of the samples was based on the method described by Jin [48]. The total RNA was isolated using TRIzol Reagent (TAKARA, Japan). Four micrograms of RNA were pretreated with RQ 1 DNAase I (Promega, USA) to remove contaminating genomic DNA. The concentration of total RNA was measured with an Infinite^®^ 200 PRO NanoQuant device (Tecan, Austria). The quality of the extracted RNA was assessed by agarose gel electrophoresis. Two micrograms of treated total RNA extracted from the fruit was used to generate cDNA samples via random priming with Superscript III reverse transcriptase (Invitrogen, Thermo Fisher Scientific, USA). The oriental melon *18S* gene was used as an internal control to normalize for small differences in the template amounts. All 18 *CmLOX* genes were analyzed. The primers for the qRT-PCR analysis were the same as those described in a previous article [44]. The qRT-PCR was performed in a 20μL reaction volume using SuperReal PreMix Plus (SYBR Green) (Cat.FP205, Tiangen Biotech, Beijing, China) on an ABI PRISM 7500 sequence-detection system (Applied Biosystems, Thermo Fisher Scientific, USA) according to the manufacturer’s instructions. The following qRT-PCR cycling conditions were employed: 50°C for 2 min, 95°C for 10 min, and 45 cycles of 95°C for 15 s and 60°C for 1 min. All qRT-PCR experiments were performed in triplicate with different cDNAs synthesized from three biological replicates. The samples were run in triplicate on each 96-well plate. The LOX/18SrRNA ratios of all samples were related to the ratio for CB and the control treatment in two different experiments, which were each set to 1. For each sample, a Ct (threshold sample) value was calculated from the amplification curves by selecting the optimalΔRn (emission of reporter dye over starting background fluorescence) in the exponential portion of the amplification plot. The relative fold differences were calculated based on the comparative Ct method using the *18S* rRNA DNA fragment of melon as an internal standard. To determine the relative fold differences for each sample in each experiment, the Ct values for all *CmLOX* mRNAs were normalized to the Ct value for 18S rRNA and calculated using the formula 2^-ΔΔCt^. The mean *CmLOX* expression levels were calculated from three biological replicates, which were obtained from three independent experiments.

### Fruit disk experiments

The methodology for fruit disk experiment was based on Zhang with modifications [42]. To determine the role of LOXs in the production of aroma compounds in highly aromatic melons, YMR samples were subjected to fruit disk experiments, which tested the effects of LOX substrates and inhibitors on the production of aroma compounds, LOX activity and expressions of the 18 *CmLOX* genes. Disks were prepared from 45 fruits. Cylinders of flesh were prepared using an 8-mm diameter cork borer, and 2 mm thick disks were cut from these flesh. The skin, seeds, and core tissues were excluded. The disks were briefly rinsed with sterile water to remove intercellular material and stored in 0.4 M mannitol until transferred to experimental treatments. For each treatment, three replicates of 30 disks were placed into 150-mL conical flasks containing 50 mL of the treatment solutions. The flesh disks were treated with 1.0 mM linoleic acid (LA), 0.5 mM LeA (linolenic acid), 0.1 mM n-propyl gallate (*n*-PG), or 0.1 mM nordihydroguariaretic acid (NDGA) in 0.4 M mannitol. Disks treated with 0.4 M mannitol alone were used as the control. Throughout treatment, the disks were shaken on a shaker in the treatment solutions for 12 h at 100 rpm and 28°C. After 12 h, the disks were blotted on filter paper, frozen in liquid nitrogen, and stored at -80°C until use to determine the total LOX activity, content of volatile compounds, and expression of 18 *CmLOX* genes.

### Statistical analysis

The experiments were performed using a completely randomized design. A principal component analysis (PCA) was employed to cluster the four aromatic varieties of melon according to their volatile composition using the SPSS 19.0 statistical software package. Other data were analyzed via analysis of variance (ANOVA) using the SPSS 19.0 statistical software package, and significant differences according to a one-way ANOVA followed by Duncan’s multiple range tests for each experiment were identified at the P < 0.05 level. The charts were generated using the Origin software package (version 8.0).

## Results

### Fruit weight, firmness, soluble solids content (SSC) and rind color

**Fig 1:**
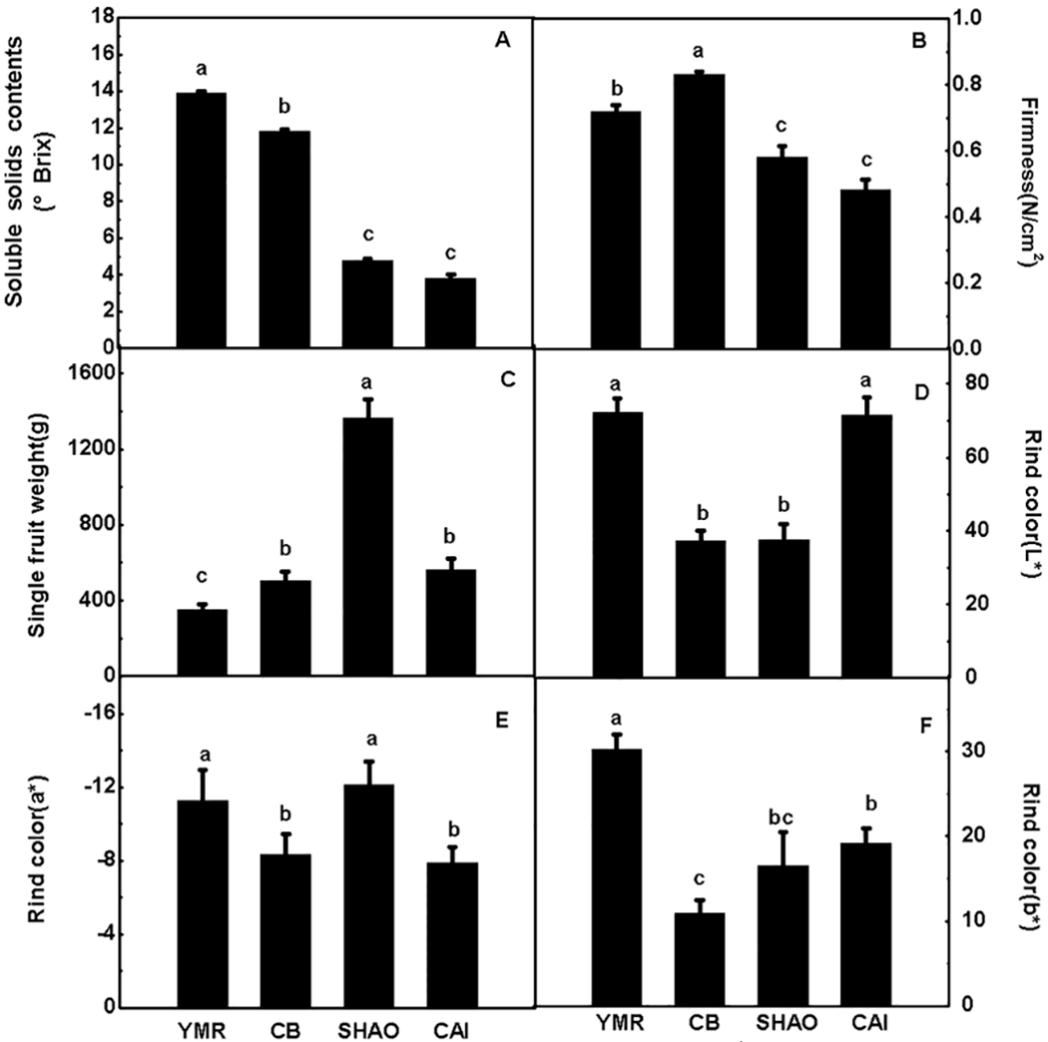
Physical signs on different aroma types of melon at their commercial maturity period. (A) Soluble solids content, (B) firmness, (C) per fruit weight, (D, E and F) pericarp color. The four types of melon included “Yu Meiren” (YMR), “Cui Bao” (CB); “Shao Gua” (SHAO) and “Cai Gua” (CAI). Duncan’s multiple range tests have been performed with different letters above the columns represent significant differences (P<0.05) between different types of melon.

To determine the maturation period, we determined the SSCs of the four types of melon fruits at different days after pollination (DAP). The SSCs directly correlated with fruit development and were maximized at 32 DAP in YMR (13.83%), 32 DAP in CB (11.8%), 28 DAP in SHAO (4.3%), and 28 DAP in CAI (3.67%). The SSCs remained stable thereafter, thus indicating that all four types of melons had reached commercial maturity (Fig 1A and S1 Fig). The SSCs of YMR and CB were similar and thrice that of SHAO and CAI. The firmness was the highest in CB, reaching 0.8 N/cm^2^; the firmness values of YMR, SHAO and CAI were 0.7, 0.55 and 0.45 N/cm^2^, respectively (Fig 1B). SHAO fruits were the heaviest, weighing 1363 g (Fig 1C). The average single-fruit weights of the other types of melon (351, 502, and 561 g) were significantly less than that of SHAO. The rind colors of YMR and CAI were brighter than those of CB and SHAO (Fig 1D, 1E and 1F). YMR was light kelly green, CB was dark green, SHAO was striped green and yellow, and CAI was light yellowish white. In conclusion, the YMR, CB, SHAO and CAI fruits exhibited different morphological and physical characteristics during maturation.

### VOC and total LOX activity analysis in different aromatic types of melon

**Fig 2:**
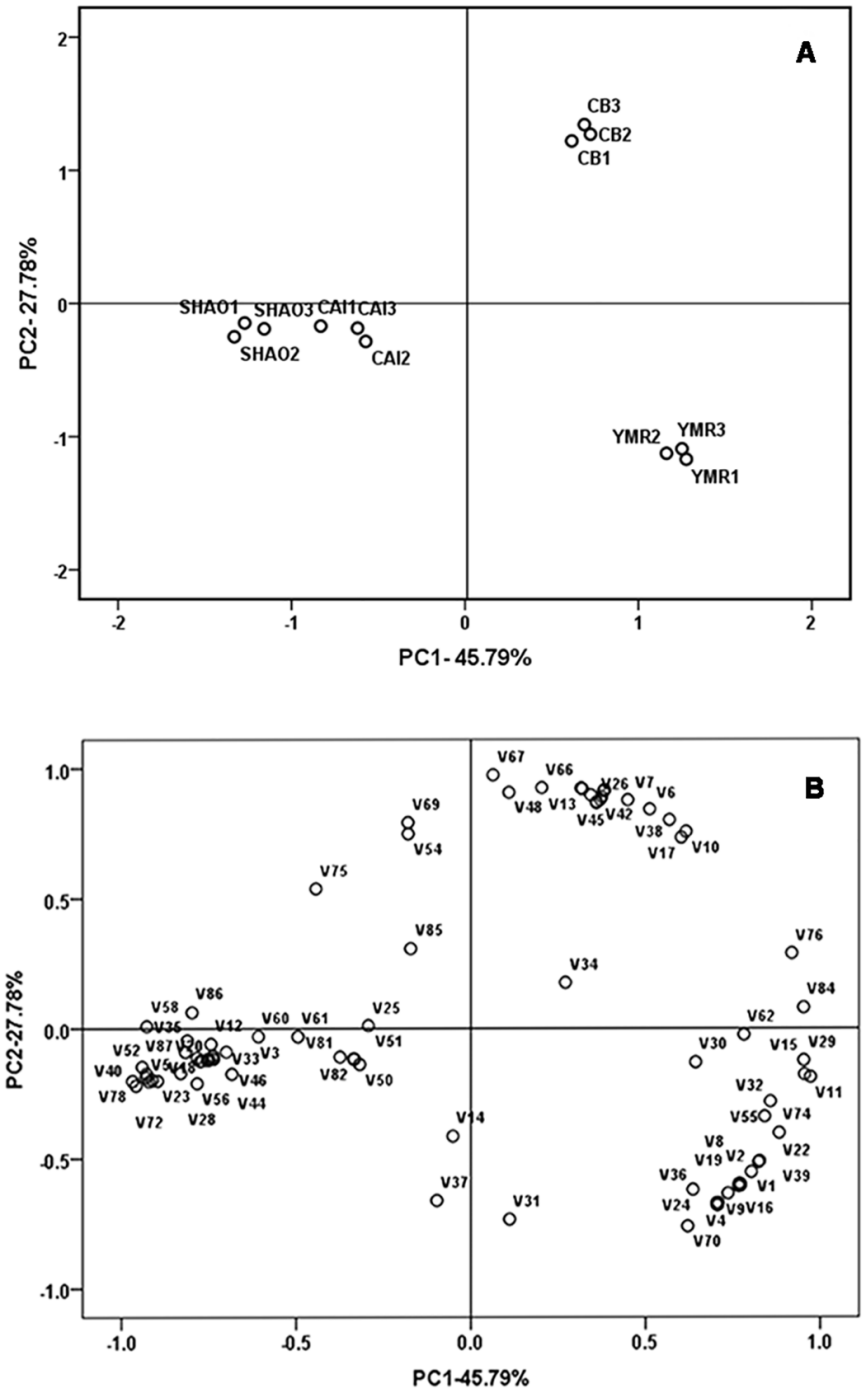
Principal component analysis (PCA) of the aroma volatiles identified in four types of melon at the commercial mature period. The four types of melon included “Yu Meiren” (YMR), “Cui Bao” (CB), “Shao Gua” (SHAO) and “Cai Gua” (CAI). (A)Scores plots of the two main principal component analysis (PCA) of the aroma volatiles identified in four types of melon at the commercial mature period. (B) Loading plots of the two main principal component analysis (PCA) of the aroma volatiles identified in four types of melon at the commercial mature period. Each sample consisted of three replicates. Codes were corresponding to the volatile compounds number in S1 Table.

**Fig 3:**
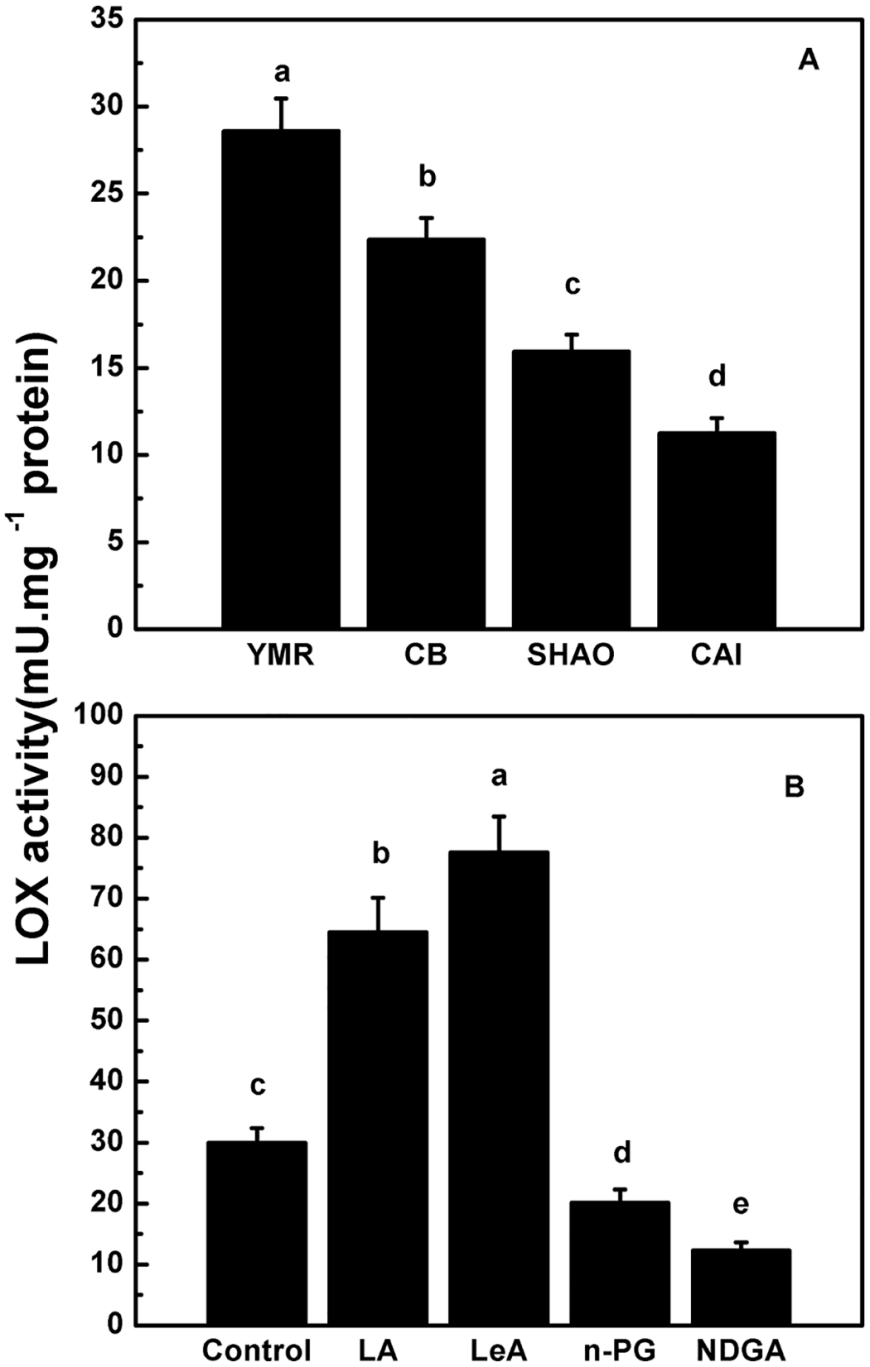
LOX activities in different types of melon and oriental melon flesh tissue disks (A and B). (A) LOX enzyme activity in flesh of different aroma types of melon. The four types of melon at the commercial mature period included “Yu Meiren” (YMR), “Cui Bao” (CB); “Shao Gua” (SHAO) and “Cai Gua” (CAI). (B) LOX enzyme activity in oriental melon flesh tissue disks. Disks were treated with 1.0 mM linoleic acid (LA), 0.5 mM linolenic acid (LeA), 0.1 mM n-propyl gallate (n-PG), or 0.1 mM nordihydroguariaretic acid (NDGA) in 0.4 M mannitol, for 12 h at 28°C, respectively. Disks treated with 0.4 M mannitol alone were used as a control. Duncan’s multiple range tests have been performed with different letters above the columns represent significant differences (P<0.05) between different types of melon.

Approximately 87 volatile compounds, including esters, aldehydes, alcohols and acids, were detected in these four types of melons (S1 Table). The total aroma compounds content (approximately 214.37 μg.g^−1^FW) and ester content (approximately 154.96 μg.g^−1^FW) in YMR were the highest among the different types of melon (Table 1). In order to visualize the differences among the four types of melon, principal component analysis was conducted to analyze these 87 compounds (Fig 2). The first two principal components accounted for 73.06% of the total variability. Fig 2A clearly shows that SHAO and CAI separated from YMR and CB across PC1. This was driven by the higher concentrations of esters such as benzyl acetate (V11) and ethyl acetate (V15). The volatile compounds associated with SHAO and CAI were mainly alcohols and aldehydes such as (*E*, *Z*)-2, 6-nonadienal (V40). CB was separated from YMR across PC2 which was affected by relative concentrations of esters and alcohols (V13, V26, V48, V66 and V67) (Fig 2B, S1 Table). Ethyl acetate, benzyl acetate (*E*, *Z*)-2, 6-nonadienal and menthol were four principal volatile compounds that affected the flavor of these four types of melon. Fig 3A shows that the total LOX activity was highest in YMR, almost twice that of SHAO and CAI. This result is consistent with the content of total aroma aromatic compounds in different types of melon (Table 1).

**Table 1:** Total and different classes of volatile compounds and their concentrations in different aromatic melon types.

Volatile compounds (μg.g^−1^FW)	Different types of melon			
	YMR	CB	SHAO	CAI
**Total ester**	154.96±14.53a	89.28±7.89b	33.29±3.90c	23.46±4.32c
**Total aldehyde**	9.42±2.59c	18.01±2.99b	38.17±4.63a	43.69±4.01a
**Total alcohol**	16.18±2.21d	43.94±4.23b	62.84±5.06a	24.07±3.94c
**Total acid**	33.81±2.61a	22.03±2.07b	22.3±4.02b	17.6±2.76b
**Total aroma**	214.37±16.51a	173.25±15.99b	156.6±6.85b	108.82±14.05c

Duncan’s multiple range tests were performed, and different letters represent significant differences (P<0.05) between different types of melon.

### Gene expression in different aromatic types of melon

**Fig 4:**
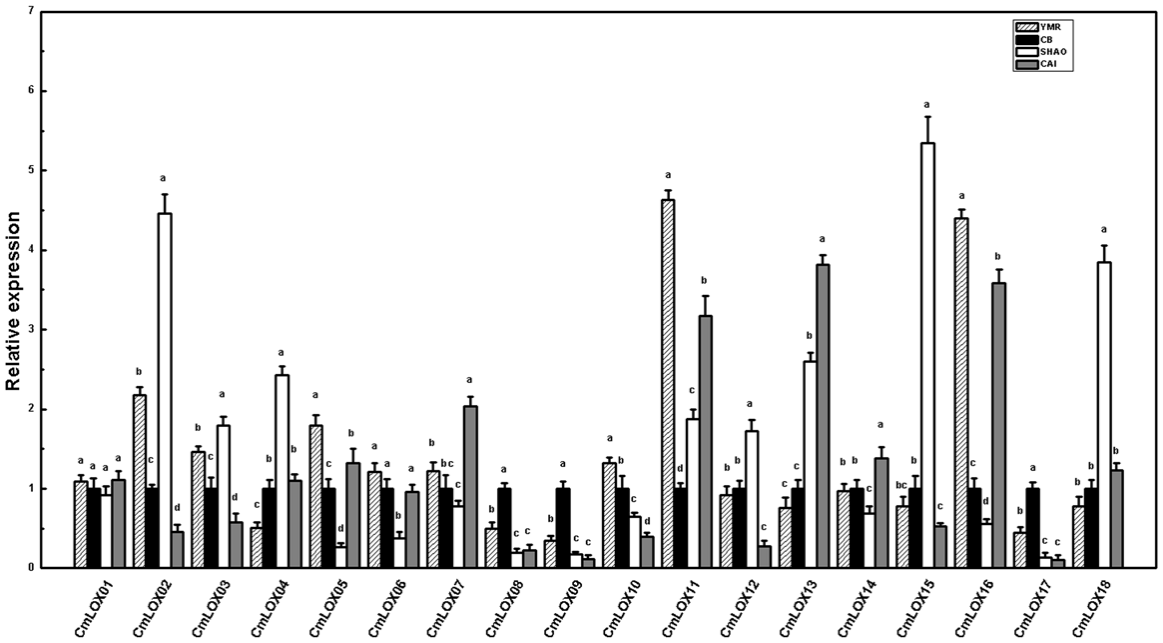
*CmLOX*s gene expression of different aroma types of melon at mature period. All of the data for LOX gene expression are means ±SE of three replicates. Expression levels of each gene are expressed as a ratio relative to the LOX/18SrRNA ratios for CB, which was set to 1. Duncan’s multiple range tests have been performed with different letters above the columns represent significant differences (P<0.05) between different types of melon.

The expression patterns of all 18 *CmLOX* genes in mature melon fruit differed by aromatic type (Fig 4). The expression levels of *CmLOX05*, *CmLOX10*, *CmLOX11* and *CmLOX16* were higher in YMR than in the other melon types. The expression levels of *CmLOX08*, *CmLOX09* and *CmLOX17* were highest in CB. Six genes (*CmLOX02*, *CmLOX03*, *CmLOX04*, *CmLOX12*, *CmLOX15* and *CmLOX18*) were highly expressed in SHAO. *CmLOX07*, *CmLOX13* and *CmLOX14* were highly expressed in CAI. Moreover, the expression of *CmLOX06* was lower in SHAO than in other types of melon, and the expression of *CmLOX01* did not significantly differ by melon type.

### VOC and total LOX activity analysis in fruit disks treated with LOX substrates and inhibitors

**Fig 5:**
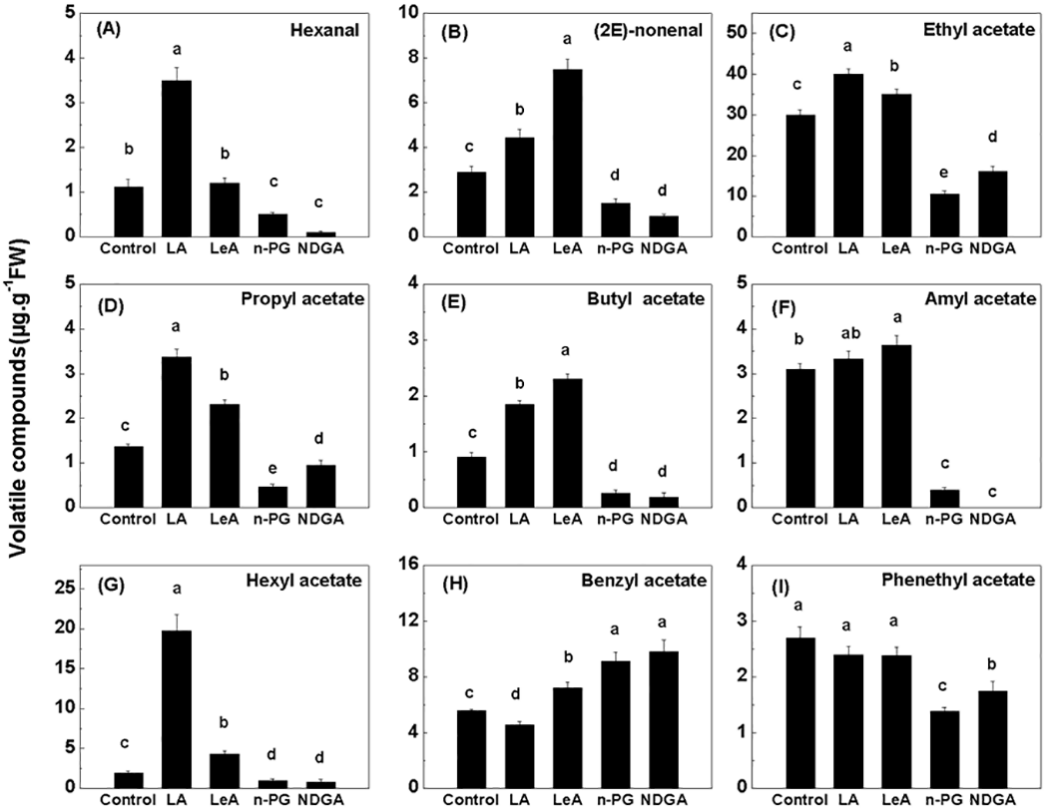
Production of volatile compounds in in oriental melon flesh tissue disks. Production of (A) hexanal, (B) (2E)-nonenal, (C) ethyl acetate, (D) propyl acetate, (E) butyl acetate, (F) amyl acetate, (G) hexyl acetate, (H) benzyl acetate and (I) phenethyl acetate in oriental melon flesh tissue disks. Disks were treated with 1.0 mM linoleic acid (LA), 0.5 mM linolenic acid (LeA), 0.1 mM n-propyl gallate (n-PG), or 0.1 mM nordihydroguariaretic acid (NDGA) in 0.4 M mannitol, for 12 h at 28°C, respectively. Disks treated with 0.4 M mannitol alone were used as a control. Duncan’s multiple range tests have been performed with different letters above the columns represent significant differences (P<0.05) between different treatments.

**Fig 6:**
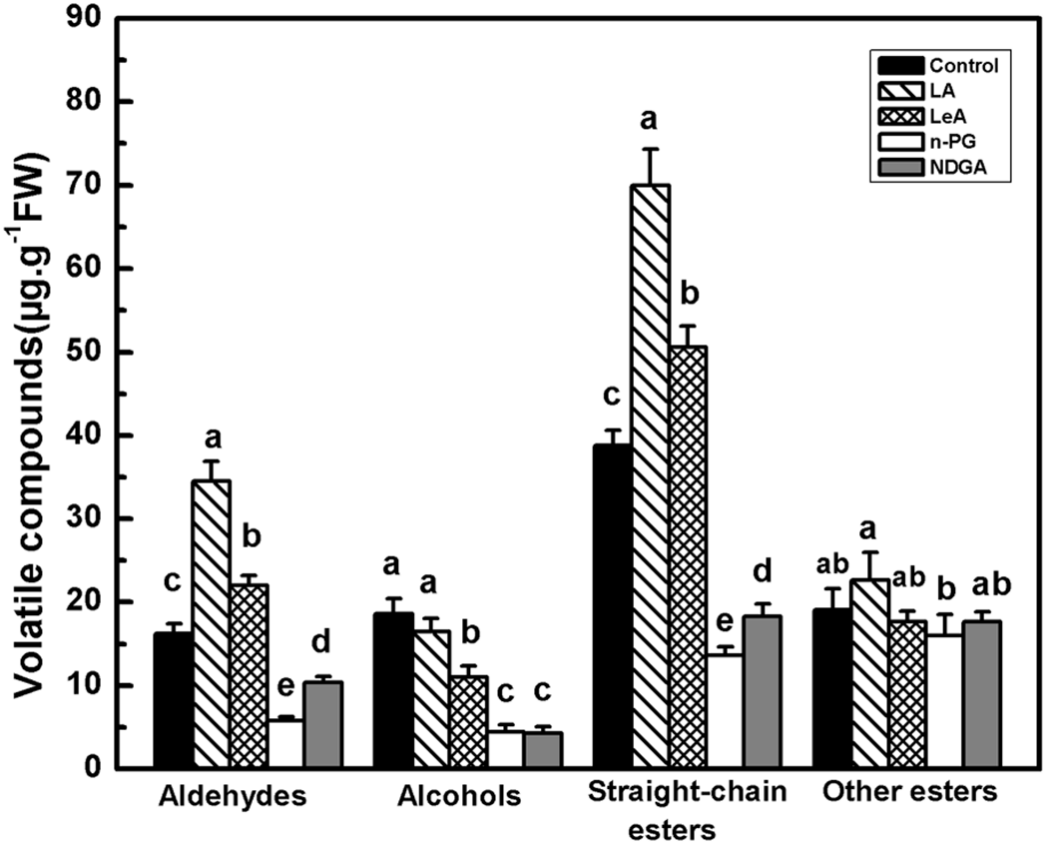
Production of aldehydes, alcohols, straight-chain esters and other esters in oriental melon flesh tissue disks. Disks were treated with 1.0 mM linoleic acid (LA), 0.5 mM linolenic acid (LeA), 0.1 mM n-propyl gallate (n-PG), or 0.1 mM nordihydroguariaretic acid (NDGA) in 0.4 M mannitol, for 12 h at 28°C, respectively. Disks treated with 0.4 M mannitol alone were used as a control. Duncan’s multiple range tests have been performed with different letters above the columns represent significant differences (P<0.05) between different treatments.

A total of 22 types of esters were evaluated via various disk treatments (S2 Table). Sixteen types of esters were detected in the control, and twenty and eighteen types of esters were detected in LA and LeA (two LOX substrates) treatments. The total volatile ester content was the highest in response to the LA treatment (approximately 94.61 μg.g^−1^ FW), which was approximately three times higher than that elicited by both *n*-PG and NDGA treatment (two LOX inhibitors). Only twelve types of esters were detected in *n*-PG and NDGA treatments. We found that treating fruit disks with LA significantly increased the production of hexanal and (2*E*)-nonenal, which are C-6 and C-9 volatile aldehydes. Compared with the control, the LeA treatment resulted significantly increased the (2*E*)-nonenal content but did not increase the production of hexanal. Conversely, addition of the two inhibitors significantly reduced the production of (2*E*)-nonenal and hexanal (Fig 5A and 5B). The treatment of fruit disks with LA and LeA (two LOX substrates) resulted in increases in the production of ethyl acetate, propyl acetate, butyl acetate, and hexyl acetate. Only the LeA treatment increased the production of amyl acetate. In contrast, *n*-PG and NDGA (inhibitors) both reduced the production of these VOCs (Fig 5C–5G). These data confirm the specific effect of substrates, such as LA, on the relevant end products, such as propyl acetate and hexyl acetate. However, the treatment of fruit disks with LA resulted in a decrease in the production of benzyl acetate, whereas the LeA, *n*-PG and NDGA treatments increased the production of this compound (Fig 5H). Neither LeA nor LA increased the production of phenethyl acetate, and both inhibitors decreased the production of this compound (Fig 5I). Substrate treatment significantly increased the production of total aldehydes and straight-chain esters. However, none of the four treatment conditions affected the total production of other esters (Fig 6).

The LA and LeA treatments supplied abundant substrate for LOX enzyme reaction and total LOX activity significantly increased the by 113% and 157% compared with the control, respectively (Fig 3B). As LOX inhibitors, NDGA treatment and *n*-PG treatment significantly inhibited the total LOX activity compared with the control group. The LOX activities in response to substrates or inhibitor treatments were consistent with the production of straight-chain esters. These results suggest that LOX directly affects the synthesis of straight-chain esters rather than the synthesis of other esters in melon flesh.

### *CmLOX* gene expression in fruit disks

**Fig 7:**
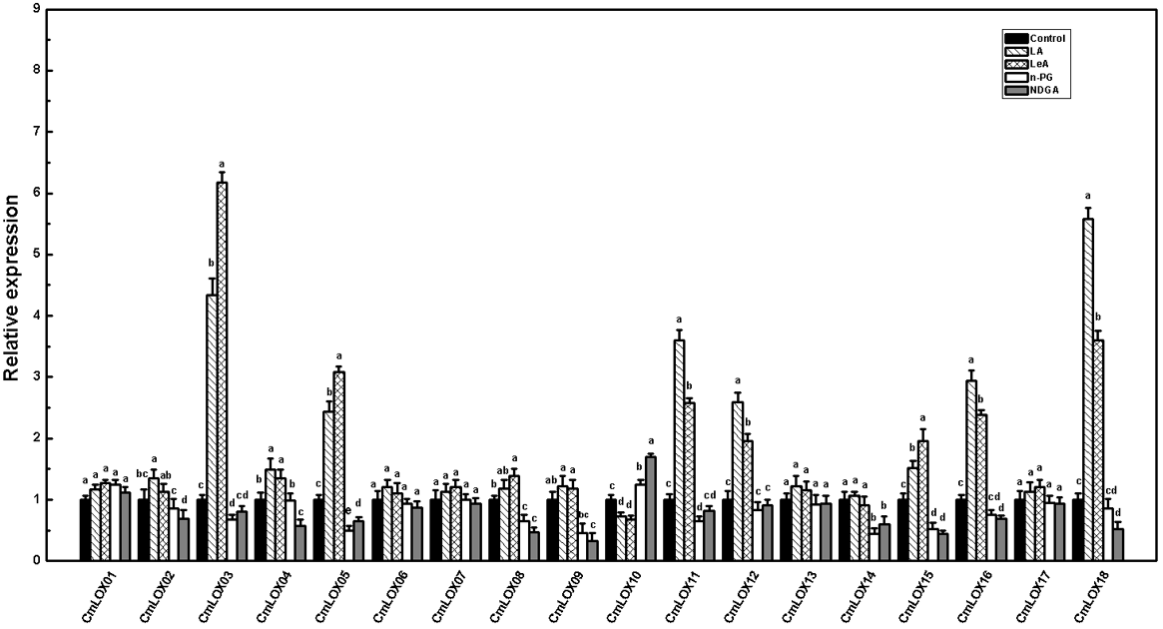
*CmLOX*s gene expression in oriental melon flesh tissue disks. Disks were treated with 1.0 mM linoleic acid (LA), 0.5 mM linolenic acid (LeA), 0.1 mM n-propyl gallate (n-PG), or 0.1 mM nordihydroguariaretic acid (NDGA) in 0.4 M mannitol, for 12 h at 28°C, respectively. Disks treated with 0.4 M mannitol alone were used as a control. All of the data for LOX gene expressions are means ±SE of three replicates. Expression levels of each gene are expressed as a ratio relative to a control, which was set at 1. Duncan’s multiple range tests have been performed with different letters above the columns represent significant differences (P<0.05) between different treatments.

Based on the fruit disk experiment, a total of 18 *CmLOX* genes were differentially expressed in the mature oriental melon (Fig 7). The expressions of *CmLOX03*, *CmLOX04*, *CmLOX05*, *CmLOX11*, *CmLOX12*, *CmLOX15*, *CmLOX16* and *CmLOX18* were substantially up-regulated by the addition of LA and LeA to the disks and down-regulated to different degrees by treatment with *n*-PG and NDGA. The *CmLOX03*, *CmLOX05* and *CmLOX15* expressions were higher in LeA treatment than that in LA, whereas *CmLOX11*, *CmLOX12*, *CmLOX16* and *CmLOX18* were up-regulated in response to the LA treatment. The transcriptional level of *CmLOX02* was up-regulated in response to the LA treatment, whereas that of *CmLOX08* was up-regulated in response to the LeA treatment. *CmLOX02* was only down-regulated by the NDGA treatment, and both inhibitors down-regulated *CmLOX08* expression. *CmLOX14* was down-regulated by addition of *n*-PG and NDGA but was not up-regulated by fatty acid treatment. Interestingly, *CmLOX09* (a 9-LOX type gene) was significantly down-regulated by the addition of *n*-PG and NDGA but was not evidently up-regulated by the addition of LOX substrates. *CmLOX10* was the only gene that was down-regulated by the addition of both LA and LeA to the disks but up-regulated by the addition of the two inhibitors. Among the other 18*CmLOX* genes, *CmLOX01*, *CmLOX06*, *CmLOX07*, *CmLOX13*, and *CmLOX17* were not responsive to the fatty acid and inhibitor treatments.

## Discussion

### VOC and LOX activity analysis

The VOC analysis enabled us to differentiate melon varieties and revealed the complex synthesis mechanism of aroma quality [49]. Specifically, the contents of aromatic compounds vary drastically according to the melon variety. Esters and aldehydes have been reported to be related to cantaloupe flavor [21]. Sulfur-containing esters and compounds with a straight six-carbon chain or a straight nine-carbon chain are present at high concentrations in cantaloupe melons and honeydew melons. Furthermore, methyl esters are abundant in Galia melons [14]. Volatile acetates are the major aroma compounds of ripening ‘Arava’ fruits, a highly aromatic melon. Volatile aldehydes and alcohols are also the most abundant aroma compounds in ‘Rochet’ fruits, a less-aromatic cultivar [18]. An aroma extract dilution analysis of the extract revealed that (*E*,*Z*)-2,6-nonadienal is the main compound of honeydew melon [20]. In our study, the morphological characteristics and physical signs in the maturation period and the type and concentration of VOCs differed according to the aromatic melon type (S1 Table). The total aroma volatile and ester contents of YMR were highest, whereas SHAO and CAI contained higher levels of alcohols and aldehydes (Table 1). The PCA indicated that SHAO and CAI were separated from YMR and CB across PC1 (Fig 2A). The primary characteristic VOC of SHAO and CAI was aldehydes such as (*E*, *Z*)-2, 6-nonadienal, so they are non-aromatic (Fig 2B). Moreover, CB was separated from YMR across PC2 (Fig 2A). The aroma profiles of YMR are mainly driven by esters and the aroma profiles of CB are mainly esters and alcohols (Fig 2B). The ester content was higher in YMR than in CB (Table 1). Thus, YMR shows highly aromatic characteristic, whereas CB shows less-aromatic characteristic. Ethyl acetate, benzyl acetate, (E, Z)-2, 6-nonadienal and menthol are four principal volatile compounds that affected the aroma of the four types of melons included in our study. These results indicated that the primary VOCs differ by melon type and that esters are important aromatic compounds in aromatic oriental melons.

Esters are derived from different pathways, and various enzymes and substrates affect their production [3]. Alcohols and aldehydes are applied as substrates to control the formation of branched-chain volatile esters in the banana [50]. However, studies of apples have found that LOX is not a key enzyme in the synthesis of aromatic compounds [27]. Furthermore, the LOX activity is poorly related to the production of volatile compounds in ‘Golden Delicious’ apple fruits for early and mid-maturity harvests. [51,52]. Benzyl acetate is the most abundant volatile molecule in the ‘Arava’ muskmelon. It is the product of the benzyl alcohol acetyl transferase-catalyzed reaction of benzyl alcohol and acetyl-CoA [53]. These types of aromatic-ring esters are derived from the amino acid pathway [19]. Straight-chain esters such as hexyl acetate and butyl acetate indicate that the LOX pathway significantly contributes to the aroma profile of apples [36,38]. Ethyl esters (especially straight-chain ethyl esters) are characteristic volatile aroma compounds of the oriental melon [24,25]. They are highly correlated with the total aroma contents. Furthermore, the production of volatile aroma compounds directly correlates with the LOX activity level [54,55]. In kiwifruit, there was an inverse relationship between total LOX activity and the accumulation of C6 aldehydes in the flesh during fruit ripening [56]. Besides, long-term controlled atmosphere storage of apple fruit caused a decrease in LOX activity corresponding with the decrease in volatiles compounds[57,58]. It suggested that negative correlation may exist between LOX activity and aldehydes accumulation. Interestingly, content level of (*E*, *Z*)-2, 6-nonadienal were higher in SHAO and CAI than other types, whereas in spite of lower LOX activity of them (Fig 3A, and S1 Table). It seems that an excess accumulation of LOX products may cause feed-back inhibition of the enzyme activity[57]. In the present study, we observed that LOX activity was highest in YMR compared with the other three melon varieties. YMR is a highly aromatic oriental melon that contains highest levels of esters, particularly straight-chain esters. Moreover, the LOX activity was lower in CB, SHAO and CAI, and these varieties contain lower total ester concentrations (Table 1, Fig 3A, and S1 Table). These results suggest that LOX activity and ester production are positively correlated in melon.

In the soybean, C6 or C9 aldehydes and alcohols are the major degradation products of LA and LeA [59]. Aldehydes also accumulate in the apple and tomato in response to LA and LeA treatment [55,60]. In kiwifruit, fatty acid treatment resulted in the accumulation of *n*-hexanal and (*E*)-2-hexenal in a fruit-disk experiment, thus suggesting that kiwifruit tissues can metabolize LA and LeA into C6 aldehyde via the LOX pathway [42]. In our fruit-disk experiments, LA significantly increased the production of hexanal, and LeA treatment significantly increased the production of hexanal and (2*E*)-nonenal (Fig 5A and 5B). Furthermore, LA and LeA also increased the accumulation of total aldehydes (Fig 6). This result indicates that C6 and C9 aldehydes metabolized via the LOX pathway may be involved in ester formation in melons. More varieties and higher concentrations of volatile esters were detected upon LOX substrate treatment (S2 Table). The production of straight-chain esters such as ethyl acetate increased in response to LA and LeA treatments and decreased in response to inhibitor treatments (Fig 5C–5E and 5G). However, the production of other esters, such as benzene methyl acetate and phenyl ethyl acetate, did not change in response to LA and LeA treatments (Figs 5H–5I and 6), likely because these aromatic ring esters are derived from the amino acid pathway [61]. Products derived from the amino acid pathway are known to be unaffected by LOX activity [3]. Thus, our results indicate that LOX does not affect the production of branched-chain esters. Our findings also suggest that LOX activity positively correlates with the straight-chain ester contents in oriental melon.

### *CmLOX* gene expression analysis

To date, a variety of LOX gene family members have been identified in plants. Their expression patterns usually differ, and some are involved in the production of volatile aroma compounds [62–66]. In kiwifruit, *AdLox1* and *AdLox5* may participate in the formation of fruit aroma [67]. In peach, *PpLOX1* and *PpLOX4* are associated with the synthesis of lactone, and *PpLOX2* and *PpLOX3* may be associated with the synthesis of C6 aldehydes [29,68]. In tobacco, *NaLOX2* is involved in the biosynthesis of 13–HPO, hexanal, and (*Z*)-hexene aldehyde [43]. *MdLOX1a* and *MdLOX5e* have been identified as candidate genes involved in the production of volatile components associated with fruit aroma in apple [66]. In pears, the expression levels of 18 LOX genes correspond to changes in the levels of volatile components [69]. Previous work demonstrated that *CmLOX01*, *CmLOX03* and *CmLOX18* are likely associated with several events during late fruit development, such as fruity aroma production [44]. In our current investigations, we observed that *CmLOX05*, *CmLOX10*, *CmLOX11* and *CmLOX16* were highly expressed in YMR, thereby suggesting that these genes might be associated with higher LOX activity in highly aromatic melons. The expression levels of *CmLOX02*, *CmLOX03*, *CmLOX04*, *CmLOX12*, *CmLOX15*, and *CmLOX18* were highest in SHAO, thus indicating that these 6 genes may be important for the synthesis of alcohols via the LOX pathway in less aromatic melons (Fig 4). The disk experiments provide a new set of data that confirm the differentiation of the *CmLOX* genes and demonstrate them to be susceptible to substrate stimulation. In the YMR fruit-disk experiment, we observed that *CmLOX03*, *CmLOX04*, *CmLOX05*, *CmLOX11*, *CmLOX12*, *CmLOX15*, *CmLOX16* and *CmLOX18* were up-regulated by the addition of two LOX substances, and they were down-regulated to different extents by the addition of LOX inhibitors. The expression of *CmLOX02* was up-regulated by LA, and the expression of *CmLOX08* was up-regulated by LeA. This pattern suggests that LOX substrate accumulation affects *CmLOX* gene expression in melons and that *CmLOX* gene expression may positively correlate with straight-chain ester production (Figs 5–7). In addition, the highest expression level of *CmLOX15* was observed at 5 DAP [44], which indicates that *CmLOX15* may play a limited role in ester production. Furthermore, ester production may be primarily due to *CmLOX03*, *CmLOX05*, *CmLOX11*, *CmLOX12*, *CmLOX16* and *CmLOX18*, especially straight-chain ester production in melon. To verify the exact roles of specific *CmLOX*s in the production of aroma compounds in melon, further studies using recombinant proteins or genetically modified technology should be conducted in the future.

## Conclusions

We identified volatile esters, especially straight-chain esters, as important VOCs in aromatic melons. The highly aromatic melon YMR contains highest concentrations of volatile esters (especially straight-chain esters) and exhibits highest LOX enzyme activity among four aroma types of melon. The application of LOX substrates increased the LOX activity and straight-chain ester concentrations. In contrast, LOX inhibitor treatment decreased these parameters. This result indicated that the LOX activity positively correlates with the straight-chain ester concentration in melons. Based on the expression patterns of 18 *CmLOX* genes in different aromatic melon types and in the fruit-disk experiment, we deem *CmLOX03*, *CmLOX05*, *CmLOX11*, *CmLOX12*, *CmLOX16* and *CmLOX18* are important candidate genes involved in straight-chain ester production in melon. Our study provides a strong support for investigating the effects of *CmLOX* genes on VOC production in melon.

## Supporting Information

S1 Fig Soluble solids content (SSC) in different aroma types of the melon ripening fruit. Include “Yu Meiren” (YMR), “Cui Bao” (CB); “Shao Gua” (SHAO) and “Cai Gua” (CAI) during their fruit ripening.
(TIF)

S2 Fig Different appearance of different aroma types of melon (*Cucumis melo*). (A)Oriental melon (*C*.*melo* var. *makuwa* Makino) cultivar “Yu Meiren”(YMR), (B)oriental melon (*C*.*melo* var. *makuwa* Makino) cultivar “Cui Bao”(CB), (C)oriental pickling melon (*Cucumis melo* var. conomon) “Shao Gua”(SHAO), (D)snake melon (*Cucumis melo L*. *var*. *flexuosus Naud)* “Cai Gua”(CAI)
(TIF)

S1 Table Volatile compounds and their concentrations (μg.g-^1^FW) in different aroma types of the melon ripen fruit. Include “Yu Meiren” (YMR), “Cui Bao” (CB); “Shao Gua” (SHAO) and “Cai Gua” (CAI). Each experiment was performed in triplicate and the mean value of their concentrations were shown in this table.
(DOCX)

S2 Table Volatile ester compounds in oriental melon flesh tissue disks. Flesh disks were treated with 1.0 mM linoleic acid (LA), 0.5 mM linolenic acid (LeA), 0.1 mM n-propyl gallate (n-PG), or 0.1 mM nordihydroguariaretic acid (NDGA) in 0.4 M mannitol, for 12 h at 28°C, respectively. Disks treated with 0.4 M mannitol alone were used as a control.
(DOCX)

S3 Table Primers of 18 *CmLOX* genes were used for qPCR analysis. (DOCX)
