# Supplementary figures and images for: The Effect of CmLOXs on the Production of Volatile Organic Compounds in Four Aroma Types of Melon (Cucumis melo)
Source: PLoS One. 2015 Nov 24;10(11):e0143567. doi: 10.1371/journal.pone.0143567 (PMC4657985; doi:10.1371/journal.pone.0143567)

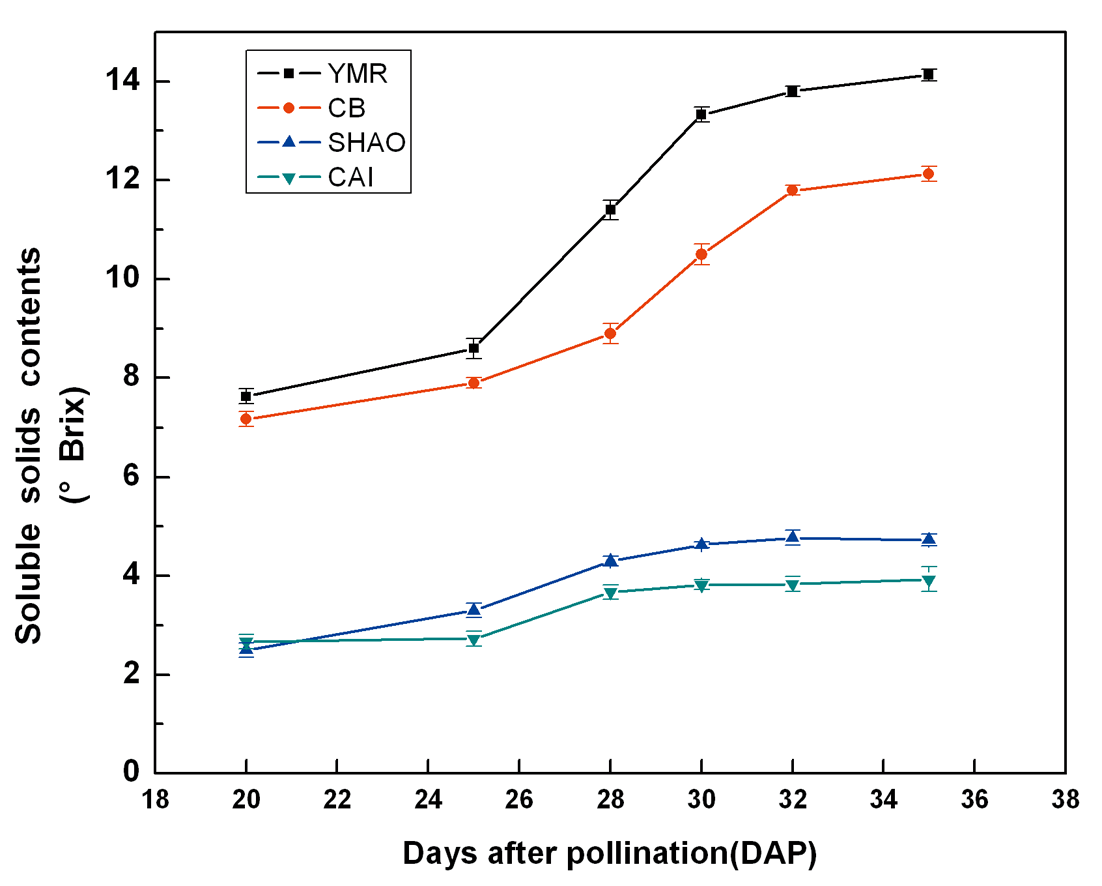

Supplement: S1 Fig — Include “Yu Meiren” (YMR), “Cui Bao” (CB); “Shao Gua” (SHAO) and “Cai Gua” (CAI) during their fruit ripening. (TIF) [file pone.0143567.s001.tif]

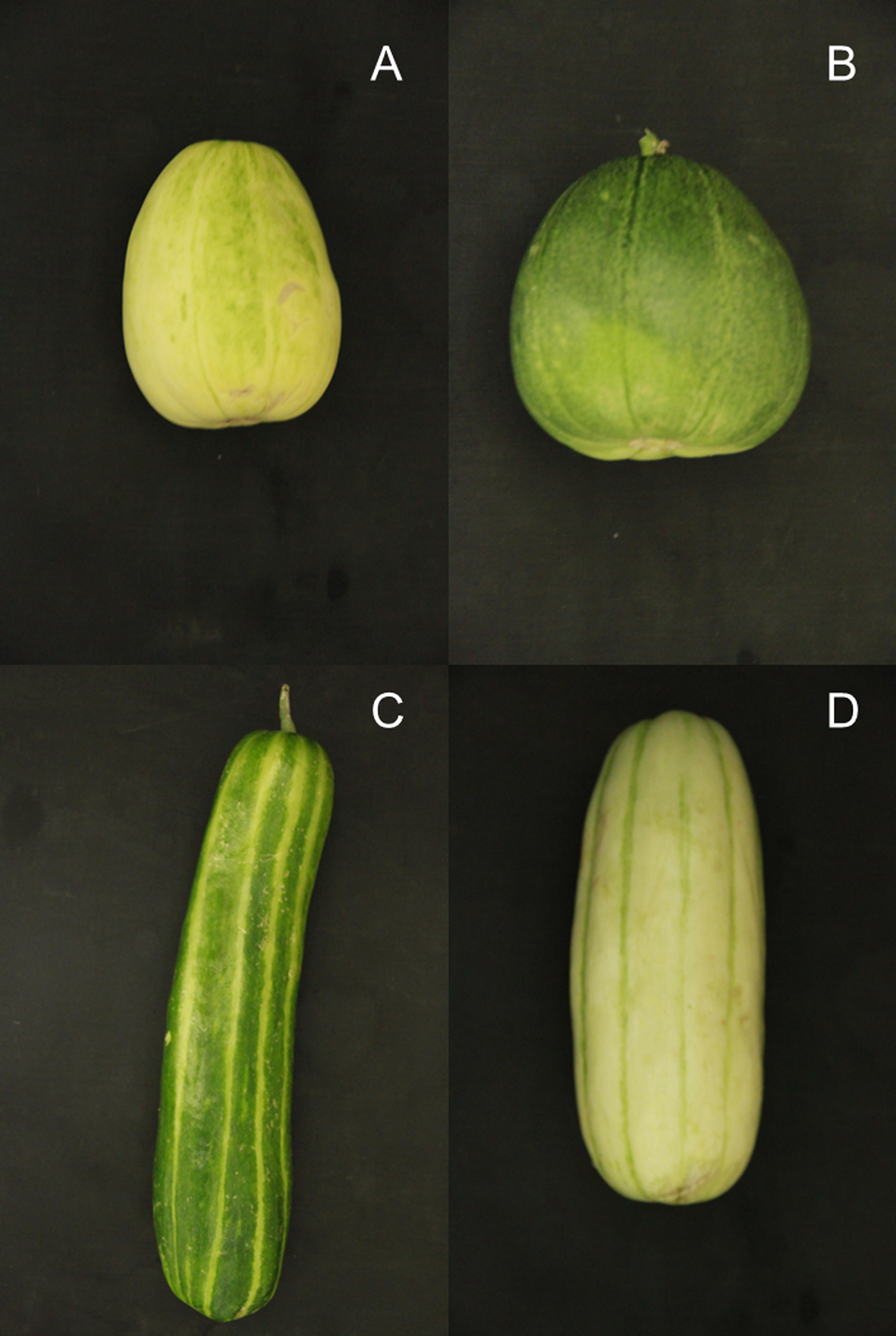

Supplement: S2 Fig — (A)Oriental melon (C.melo var. makuwa Makino) cultivar “Yu Meiren”(YMR), (B)oriental melon (C.melo var. makuwa Makino) cultivar “Cui Bao”(CB), (C)oriental pickling melon (Cucumis melo var. conomon) “Shao Gua”(SHAO), (D)snake melon (Cucumis melo L. var. flexuosus Naud) “Cai Gua”(CAI) (TIF) [file pone.0143567.s002.tif]
