# Supplementary material for: The Effect of CmLOXs on the Production of Volatile Organic Compounds in Four Aroma Types of Melon (Cucumis melo)
Source: PLoS One. 2015 Nov 24;10(11):e0143567. doi: 10.1371/journal.pone.0143567 (PMC4657985; doi:10.1371/journal.pone.0143567)
Supplement: S1 Table — Include “Yu Meiren” (YMR), “Cui Bao” (CB); “Shao Gua” (SHAO) and “Cai Gua” (CAI). Each experiment was performed in triplicate and the mean value of their concentrations were shown in this table. (DOCX) [file pone.0143567.s003.docx]

**S1 Table**

| **Volatile compounds**  **(μg.g^-1^FW)** | | | **Different types of melon** | | | |
| --- | --- | --- | --- | --- | --- | --- |
|  |  |  | **YMR** | **CB** | **SHAO** | **CAI** |
| V1 | 1-Methylbutyl acetate | | 7.2 | 0.7 | ND | ND |
| V2 | | 2-Methylbutyl acetate | 17.2 | 2.8 | ND | ND |
| V3 | | 2-Propenoic acid, 2-methyl-, 1-methylbutyl ester | 0.42 | 0.52 | 5.33 | ND |
| V4 | | 4-Hexen-1-ol, acetate | 0.13 | ND | ND | ND |
| V5 | | 5-Isopropenyl-2-methylcyclohexyl acetate | ND | ND | 0.33 | 0.21 |
| V6 | | 8-Nonynoic acid, methyl ester | 0.45 | 2.69 | ND | ND |
| V7 | | Acetic acid, (1-methylethoxy)-, ethyl ester | 0.88 | 7.18 | 0.26 | 0.44 |
| V8 | | Allyl acetate | 12.27 | 1.25 | ND | ND |
| V9 | | Allyl acrylate | 0.12 | ND | ND | ND |
| V10 | | Amyl acetate | 4.07 | 14.22 | ND | ND |
| V11 | | Benzyl acetate | 28.08 | 19.67 | 6.8 | 13.22 |
| V12 | | Cyclopropanecarboxylic acid, cyclohexyl ester | ND | 0.36 | 5.33 | ND |
| V13 | | Cyclopropylmethanol acetate | ND | 3.5 | 0.33 | ND |
| V14 | | Diallyl oxalate | 0.17 | ND | ND | 0.42 |
| V15 | | Ethyl Acetate | 37.14 | 24.22 | 4.2 | 3.7 |
| V16 | | Ethyl caproate | 2.05 | 0.22 | ND | ND |
| V17 | | Ethyl linoleate | 0.87 | 2.85 | ND | ND |
| V18 | | Ethyl methyl carbonate | ND | ND | 1.93 | 1.67 |
| V19 | | Ethyl pyruvate | 8.64 | 0.55 | 0.13 | ND |
| V20 | | Formic acid isopropyl ester | ND | ND | 0.44 | ND |
| V21 | | Hexyl acetate | 7.89 | 1.1 | 0.28 | 0.41 |
| V22 | | Isobutyl acetate | 10.27 | 2.15 | 0.1 | ND |
| V23 | | Isobutylnitrite | ND | ND | 0.66 | 0.79 |
| V24 | | Isopropyl acetate | 0.36 | ND | ND | ND |
| V25 | | Leaf acetate | ND | 0.11 | ND | 0.79 |
| V26 | | Methyl 14-(2-Octylcyclopropyl)Tetradecanoate | ND | 0.27 | ND | ND |
| V27 | | Methyl hexadecanoate | 10.98 | 1.3 | 0.25 | ND |
| V28 | | Monobutyl phthalate | ND | ND | 2.62 | 0.07 |
| V29 | | n-Propyl acetate | 0.57 | 0.33 | ND | 0.07 |
| V30 | | Oxalic acid, allyl nonyl ester | 0.12 | 0.08 | 0.06 | ND |
| V31 | | Oxalic acid, hexadecyl hexyl ester | 0.12 | ND | 0.09 | ND |
| V32 | | Phenethyl acetate | 4.6 | 2.7 | 1.52 | 0.86 |
| V33 | | Phthalic acid, cyclohexyl 2-pentyl ester | ND | ND | 2.62 | ND |
| V34 | | Propargyl acetate | 0.37 | 0.51 | ND | 0.81 |
| V35 | | Vinyl formate | ND | ND | 3.81 | ND |
| V36 | Trans-2-hexenyl | | 0.14 | ND | ND | ND |
| V37 | 4-Pentenal | | 0.55 | ND | 0.6 | ND |
| V38 | | Hexanal | 1.2 | 2.35 | 0.81 | 1.03 |
| V39 | | (2*E*)-nonenal | 3.2 | 0.68 | ND | ND |
| V40 | | (*E*,*Z*)-2,6-nonadienal | 3.78 | 13.78 | 26.65 | 41.39 |
| V41 | | 3,6-nonadienal | 0.56 | 0.88 | 6.31 | 1.27 |
| V42 | | 1,3-Dimercaptopropane | ND | 0.33 | ND | ND |
| V43 | | 1-Hepten-4-ol | ND | ND | 0.61 | 0.29 |
| V44 | | 1-Pentanol | ND | ND | 0.11 | 0.27 |
| V45 | 2,4-Dimethyl-3-hexanol | | ND | 1.04 | ND | ND |
| V46 | | 2,4-Pentanediol | ND | ND | 1.47 | ND |
| V47 | | 2-Decyn-1-ol | ND | 0.4 | ND | ND |
| V48 | | 2-Hexen-1-ol | ND | 0.54 | ND | 0.21 |
| V49 | | 2-Hexen-1-ol, 2-ethyl- | ND | 0.54 | ND | ND |
| V50 | | 2-Hexyn-1-ol | 0.37 | ND | ND | 12.92 |
| V51 | | 2-Methyl-1-propanol | ND | ND | ND | 0.77 |
| V52 | | 2-Methyl-5-Hexen-3-ol | ND | ND | 0.71 | 0.28 |
| V53 | | 2-Nitro-1-butanol | ND | ND | 0.42 | ND |
| V54 | | 2-Nonyn-1-ol | 1.87 | 12.8 | 10.69 | 1.02 |
| V55 | | 2-Nonen-1-ol | 5.02 | 2.16 | 0.79 | ND |
| V56 | | 2-Octyn-1-ol | 1.3 | 0.17 | 12.69 | 1.8 |
| V57 | | 3,4-Dimethyl-5-Hexen-3-ol | ND | ND | 5.95 | ND |
| V58 | | 3-Buten-2-ol | ND | 0.11 | 0.59 | 0.22 |
| V59 | | 4-Methyl-3-Penten-1-ol | 1.1 | 1.37 | 16.63 | 2.07 |
| V60 | | 4-Penten-2-ol | 0.08 | 0.11 | 0.47 | ND |
| V61 | | 5-Methyl-1-Hexyn-3-ol | ND | 0.26 | 0.45 | 2.1 |
| V62 | | Benzyl Alcohol | 0.72 | 0.55 | 0.26 | ND |
| V63 | | Borneol | ND | 1.8 | ND | ND |
| V64 | | Cis-5-octen-1-ol | 4.69 | ND | ND | ND |
| V65 | | Cis-6-Nonen-1-ol | ND | 3.05 | ND | ND |
| V66 | | Eugenol | ND | 1.35 | 0.34 | ND |
| V67 | | Menthol | ND | 2.77 | 0.98 | 0.51 |
| V68 | | Oct-1-Yn-4-ol | ND | ND | 5.33 | 0.19 |
| V69 | | Phenol, 2-methoxy-4-(1-propenyl)- | ND | 1.35 | 1.03 | ND |
| V70 | | Trans,cis-2,6-Nonadien-1-ol | 1.03 | ND | ND | 0.36 |
| V71 | | Trans,cis-3,6-Nonadien-1-ol | ND | 13.45 | 1.3 | ND |
| V72 | | Trans,trans-2,4-Hexadien-1-ol | ND | ND | 0.82 | 0.89 |
| V73 | | Trans-3-Hexen-1-ol | ND | 0.12 | 1.19 | 0.15 |
| V74 | | (Isopropylthio) Acetic acid | 2.35 | 0.77 | ND | ND |
| V75 | | 2-Oxopentanoic acid | ND | 0.03 | 0.04 | ND |
| V76 | | 4-Methylvaleric acid | 8.65 | 9.65 | 1.24 | 1.08 |
| V77 | Acetic acid, hydrazide | | ND | 0.4 | 0.02 | ND |
| V78 | | Acetoxyacetic acid | ND | ND | 4.36 | 3.44 |
| V79 | | Capric acid | 0.09 | 0.13 | 3.36 | ND |
| V80 | | cis-9-Octadecenoic acid | 10.11 | 0.17 | 0.12 | 0.11 |
| V81 | | Hendecanoic acid | ND | 0.07 | 0.21 | 4.17 |
| V82 | | Hexanoic anhydride | ND | ND | ND | 0.13 |
| V83 | | hydnocarpic acid | ND | ND | 0.85 | ND |
| V84 | | Isobutyric acid | 12.49 | 10.49 | 0.44 | ND |
| V85 | | Levulinic acid | ND | 0.11 | ND | 0.25 |
| V86 | | Myristic acid | 0.13 | ND | 6.36 | 5.72 |
| V87 | | Palmitic acid | ND | 0.21 | 5.32 | 2.71 |

**Note: ND, Not be detected.**
