# Supplementary material for: The Effect of CmLOXs on the Production of Volatile Organic Compounds in Four Aroma Types of Melon (Cucumis melo)
Source: PLoS One. 2015 Nov 24;10(11):e0143567. doi: 10.1371/journal.pone.0143567 (PMC4657985; doi:10.1371/journal.pone.0143567)
Supplement: S2 Table — Flesh disks were treated with 1.0 mM linoleic acid (LA), 0.5 mM linolenic acid (LeA), 0.1 mM n-propyl gallate (n-PG), or 0.1 mM nordihydroguariaretic acid (NDGA) in 0.4 M mannitol, for 12 h at 28°C, respectively. Disks treated with 0.4 M mannitol alone were used as a control. (DOCX) [file pone.0143567.s004.docx]

**S2 Table**

| **Volatile esters**  **(μg.g^-1^FW)** | **Treatments** | | | | |
| --- | --- | --- | --- | --- | --- |
|  | **Control** | **LA** | **LeA** | **n-PG** | **NDGA** |
| Ethyl acetate | 30.03 | 39.99 | 35.13 | 10.50 | 16.12 |
| Propyl acetate | 1.36 | 3.37 | 2.31 | 0.46 | 0.95 |
| Butyl acetate | 0.91 | 1.85 | 2.32 | 0.26 | 0.18 |
| Amyl acetate | 3.09 | 3.33 | 3.62 | 0.39 | ND |
| Hexyl acetate | 1.90 | 19.74 | 4.37 | 0.98 | 0.80 |
| Ethyl (methylthio)acetate | 0.39 | 0.17 | 0.08 | 0.26 | 0.27 |
| Benzyl acetate | 5.56 | 4.54 | 7.2 | 9.09 | 9.82 |
| Phenethyl acetate | 2.69 | 2.39 | 2.38 | 1.38 | 1.74 |
| Ethyl pyruvate | 1.12 | 1.53 | 2.74 | 0.77 | ND |
| Hexyl hexanoate | 0.15 | 0.17 | 0.10 | ND | ND |
| 2-Methylbutyl acetate | 1.79 | 1.82 | 1.91 | 1.69 | 1.75 |
| 9-Octadecen-12-ynoic acid methyl ester | 0.41 | 0.53 | 0.84 | 2.07 | 0.96 |
| Acetic acid, (1-methylethoxy)-, ethyl ester | 6.76 | 4.46 | ND | ND | ND |
| 9,15-Octadecadienoic acid, methyl ester | 0.41 | 0.35 | 0.20 | ND | 0.42 |
| 3-Phenylpropanol acetate | ND | ND | 0.15 | ND | ND |
| Hexyl methacrylate | 0.67 | 4.16 | 1.47 | ND | 2.98 |
| Benzyl carbamate | ND | ND | ND | 1.81 | ND |
| (*E*)-10-Heptadecen-8-ynoic acid methyl ester | ND | 0.08 | ND | ND | ND |
| 3-Phenylpropanol acetate | 0.61 | 0.49 | 0.145 | ND | ND |
| (*E*)-2-Hexenyl hexanoate | ND | 1.77 | 1.8 | ND | ND |
| *(E)*-2-hexenyl butanoate | ND | 1.4 | 1.5 | ND | ND |
| 2-methylallyl propionate | ND | 0.47 | ND | ND | ND |

**Note: ND, Not be detected.**
