# Supplementary material for: The Effect of CmLOXs on the Production of Volatile Organic Compounds in Four Aroma Types of Melon (Cucumis melo)
Source: PLoS One. 2015 Nov 24;10(11):e0143567. doi: 10.1371/journal.pone.0143567 (PMC4657985; doi:10.1371/journal.pone.0143567)
Supplement: S3 Table — (DOCX) [file pone.0143567.s005.docx]

**S3 Table**

| Pimer name | Sequence(5’to 3’) | Size(bp) |
| --- | --- | --- |
| *CmLOX01-F* | CCATCAACTTATCAGCCATT | 189bp |
| *CmLOX01-R* | GTTCGTTCAAGAAGACCAT |  |
| *CmLOX02-F* | TAGCACCGAAGGAATCAC | 252bp |
| *CmLOX02-R* | AGACAGCACAATAACAGAGT |  |
| *CmLOX03-F* | GACGACGAGAATGGAGAG | 157bp |
| *CmLOX03-R* | GCTGGTTGAACTGTTGATAC |  |
| *CmLOX04-F* | GCTCAGTGAAGTTATCAAGA | 143bp |
| *CmLOX04-R* | GCTCAGTGAAGTTATCAAGA |  |
| *CmLOX05-F* | GCTGCTTGTTCCTCATTA | 206bp |
| *CmLOX05-R* | AGTCTTCAACTGCCATTC |  |
| *CmLOX06-F* | GTGTATGTTCCAAGAGATG | 293bp |
| *CmLOX06-R* | TGAATAAGTTGAGGAGTA |  |
| *CmLOX07-F* | TACTTGGAGGAATGGATA | 236bp |
| *CmLOX07-R* | TTGTTGTAACGGTGAGAC |  |
| *CmLOX08-F* | GGTAACTGGTCGTGGAAT | 246bp |
| *CmLOX08-R* | AAGGCAGAGGAATAACAGAA |  |
| *CmLOX09-F* | CAGATCCATCTTGTGAAC | 230bp |
| *CmLOX09-R* | AGTTGGTAGAGTCATTCC |  |
| *CmLOX10-F* | TGACAGGACAAGGAGTTC | 183bp |
| *CmLOX10-R* | CGGTATTGGCAAGAATGTTA |  |
| *CmLOX11-F* | CAAGTCATTCTCCAGATG | 192bp |
| *CmLOX11-R* | GTTGATAAGGTCCAATCC |  |
| *CmLOX12-F* | GTTAAGTTCTTCAGCATACG | 218bp |
| *CmLOX12-R* | ACGAGGATGGATAGTAATG |  |
| *CmLOX13-F* | CAAGCAACACAGGTAATG | 205bp |
| *CmLOX13-R* | CTCCAGTTCTATTCTTCAAG |  |
| *CmLOX14-F* | CAAGTGAACCAGATAACAAG | 180bp |
| *CmLOX14-R* | CAGAGGAATTGGAATGAAG |  |
| *CmLOX15-F* | CTATTATGCTGATGCTGAG | 194bp |
| *CmLOX15-R* | GAAGGAAGTTGACAGATG |  |
| *CmLOX16-F* | ATACGGACCTCAAGAATC | 218bp |
| *CmLOX16-R* | GAGTCAAAGTGTCATCAG |  |
| *CmLOX17-F* | TGACTATCTAATGCCACTTC | 160bp |
| *CmLOX17-R* | CCAACTTATCTCTTCTCCT |  |
| *CmLOX18-F* | TGGAGACTATCACAATCG | 195bp |
| *CmLOX18-R* | CTTTCCCATCACCTCTAA |  |
